# Feature-selective adaptation of numerosity perception

**DOI:** 10.1098/rspb.2024.1841

**Published:** 2025-01-29

**Authors:** Camilla Caponi, Elisa Castaldi, Paolo Antonino Grasso, Roberto Arrighi

**Affiliations:** ^1^ Department of Neuroscience, Psychology, Pharmacology and Child Health, University of Florence, Florence, Italy; ^2^ Department of Physics and Astronomy, University of Florence, Florence, Italy

**Keywords:** numerosity perception, perceptual adaptation, numerosity detectors, adaptation selectivity, visual system

## Abstract

Perceptual adaptation has been widely used to infer the existence of numerosity detectors, enabling animals to quickly estimate the number of objects in a scene. Here, we investigated, in humans, whether numerosity adaptation is influenced by stimulus feature changes as previous research suggested that adaptation is reduced when the colour of adapting and test stimuli did not match. We tested whether such adaptation reduction is due to unspecific novelty effects or changes of stimuli identity. Numerosity adaptation was measured for stimuli matched or unmatched for low-level (colour, luminance, shape and motion) or high-level (letters' identity and face emotions) features. Robust numerosity adaptation occurred in all conditions, but it was reduced when adapting and test stimuli differed for colour, luminance and shape. However, no reduction was observed between moving and still stimuli, a readable change that did not affect the item’s identity. Similarly, changes in letters' spatial rotations or face features did not affect adaptation magnitude. Overall, changes in stimulus identity defined by low-level features, rather than novelty in general, determined the strength of the adaptation effects, provided these changes were readily noticeable. These findings suggest that numerosity mechanisms operate on categorized items in addition to the total quantity of the set.

## 1. Introduction

Humans share with other animal species the ability to make rapid and reasonably accurate estimates of the number of items without relying on serial counting [1]. This ability is present from birth [2,3], and occurs spontaneously [4–9] and universally even in individuals with no access to formal schooling [10,11]. Dedicated numerosity detectors, clearly identified in non-human animals, subserve this fundamental visual property. Electrophysiological studies found that activity of individual cells in the prefrontal and parietal cortices of macaques and in the endbrain of crows was finely tuned to the numerosities of visual arrays [12–14]. In humans, with the noticeable exception of a few electrocorticography studies [15,16], evidence of the existence of number-selective detectors mainly comes from psychophysical studies using the adaptation technique [17]. This technique can reveal neural populations that are responsive to the examined feature (e.g. motion direction, orientation and size) and provide insights into the feature’s representational and functional properties. A large body of studies found that numerosity, like many other primary perceptual features, is susceptible to adaptation which elicits aftereffects: exposing individuals to higher numerosities makes the following ones appear less numerous, suggesting the presence of dedicated neural mechanisms for the analysis of numerical quantities [18–27]. Leveraging on a similar technique, early functional magnetic resonance imaging (fMRI) habituation studies in humans recorded distance-dependent signal release from adaptation and described tuning curves for numerosity in the intraparietal sulcus (IPS) see [28].

Across all these studies, one characteristic of numerosity adaptation was that it occurred largely independently of changes in low-level features and even sensory modality, suggesting that the representation of numbers may be abstract and amodal. Indeed, fMRI studies showed that the signal release from habituation in the IPS was significantly higher for numerical deviants than for shape deviants [29] and that it was notation-independent, resulting in similar recovery of the signal irrespective of the numerical deviant being presented in non-symbolic (dot arrays) or symbolic (digits) format [30]. Psychophysical studies showed that, in addition to the visual domain, numerosity adaptation also occurs in other sensory modalities, including audition [19,25,26] and touch [31]. Importantly, several studies have shown that numerosity adaptation is generalized across modalities: adapting to auditory sequences influenced the perceived numerosity of sequences of visual stimuli and vice versa [19], and the same was observed in the tactile modality [26,31]. Furthermore, the adaptation effect is capable of generalizing across motor and sensory domains [32–34] even in the absence of visual experience [34].

While all these studies clearly point to the existence of generalized and a-modal mechanisms tuned to numerosity perception, it might also be ecologically important to have a system capable of segregating different objects within the visual scene that provides independent numerosity estimates for the items of different groups, such as edible (ripe) and not edible (unripe) fruits. Indeed, previous evidence showed that human adults can attentively encode, in parallel, different subsets of items defined by colour, suggesting that approximate numbers can be stored as a feature of each set [35]. In line with the possibility of creating parallel representation of numerosity, a recent study demonstrated that numerosity adaptation is tuned to specific features of the stimuli such as colour in vision and pitch in sound [25]. Participants were adapted to high numerosity dot arrays with items’ colour either matched to or different from the test. Numerosity underestimation (about 25%) was observed for matched colours (either physical or perceived), while the adaptation effect was much reduced when the adaptor and test colours differed, with a similar selective adaptation effect also occurring in audition.

To further investigate the phenomenon of selectivity in numerosity adaptation, the current study assessed which changes in stimulus features lead to a reduction in adaptation, and whether such a reduction is tied to changes that induce a shift in stimuli ‘identity’. Addressing this question is particularly important in light of a recent theory suggesting that what has been traditionally interpreted as an effect of adaptation on the numerosity of a set, may instead be accounted for by saliency changes at the level of individual items when stimulus characteristics are modulated between the adaptation and test phases [36,37] (but see also [38]). Here, we conducted six experiments wherein adult participants were adapted to high numerosities and tested with stimuli that either matched or differed from the adaptor in various low-level features (colour, luminance, shape and motion) or high-level characteristics (letters’ identity, face expressions). The first three experiments, which modulated colour, luminance and shape congruency between adaptor and test stimuli, aimed to replicate previous findings by Grasso *et al*. [39] and extend them to perceptual domains beyond colour. In the fourth experiment, we manipulated the motion profiles of the items (still versus randomly moving items) between adaptor and test—a feature change that creates a significant difference between stimuli without implying an identity change of the items. In other words, if numerosity adaptation selectivity depends on stimulus identity, rather than on unspecific changes in stimulus features, we might expect adaptation to moving items to produce the same adaptation magnitude whether the test stimuli are still or moving. Finally, we further investigated the role of stimulus identity in numerosity adaptation by using stimuli that allowed us to contrast high-level linguistic meaning with low-level feature differences. In detail, when using letters as stimuli, they may differ in identity due to high-level linguistic meanings, yet are defined by the same shape, simply rotated in space (e.g. 'b' versus 'p', 'q' and 'd'). Letters were then useful to test the opposite condition where the identity of the same item at the linguistic level was preserved despite major changes in the low-level characteristics of the stimuli, as seen when the letters were displayed in different notations (i.e. lowercase ‘b’ versus uppercase ‘B’). If adaptation selectivity is determined by stimulus high-level linguistic identity, we should observe the same adaptation effect for ‘b’ and ‘B’ and a reduced effect for all the other, lowercase, letters. On the contrary, if adaptation selectivity does not apply to high-level linguistic stimuli, the change in letter notation from ‘b’ to ‘B’ should elicit the weaker adaptation effect, because this pair exhibits the greatest variation in low-level features (such as shape). Finally, in a sixth experiment, the role of stimuli identity was tested via ecological stimuli such as faces (represented by smiley ideograms) with different emotional expressions (happy or sad faces) or with corrupted spatial arrangement of the inner components (scrambled faces). If adaptation selectivity is related to stimulus identity, we should observe the same adaptation effect irrespective of faces’ emotion, unless these are coded as different individuals (e.g. enemies versus friends). On the contrary, we expect a decreased adaptation for scrambled compared to non-scrambled faces since they should be perceived as belonging to different classes of stimuli. To anticipate the results, we found that stimulus identity, as defined by low-level features, is the crucial factor in determining the magnitude of numerosity adaptation aftereffects, while complex high-level characteristics pointing to linguistic or emotional cues failed or were much less effective in modulating the magnitude of adaptation aftereffects. Using a model of the primary visual cortex response to different classes of stimuli used across our experiments, we measured the low-level ‘dissimilarity’ between adaptor and test stimuli and found that such parameters strongly affected the adaptation magnitude, with higher dissimilarity being associated with weaker adaptation aftereffects.

## 2. Methods

### (a) Participants

The sample size was calculated using an *a priori* power analysis (G*Power software, version 3.1 [40]). The effect size was estimated from a study by Grasso *et al.* [39] that exploited very similar methods. With an *⍺* = 0.05 and a power of 0.9, the analysis suggested a required sample size of 18 participants.

Across all experiments, a total of 56 participants took part in the study (mean age: 25.2 ± 3.9, 37 females). Each participant performed one or more of six different experiments which differed in the type of feature characterizing the arrays of visual stimuli as detailed below. All participants had normal or corrected-to-normal visual acuity and provided written informed consent prior to the experiment.

#### (i) Stimuli and procedure

Participants sat 57 cm away from a 27″ screen monitor (resolution 2560 × 1440 pixels; refresh rate 60 Hz), in a quiet and dimly lit room.

Across six experiments we tested the selectivity of numerosity adaptation to different classes of stimuli where adapting and adapted items were congruent or not congruent for colour (experiment 1), luminance (experiment 2), shape (experiment 3) and motion (experiment 4). Two additional experiments were carried out to test selective adaptation for conditions in which stimuli identity prompted a linguistic recognition process such as letter identification (experiment 5) and discrimination of emotional state of stylized faces or arrangement of the local inner components (faces versus scrambled faces; experiment 6).

**Figure 1.**
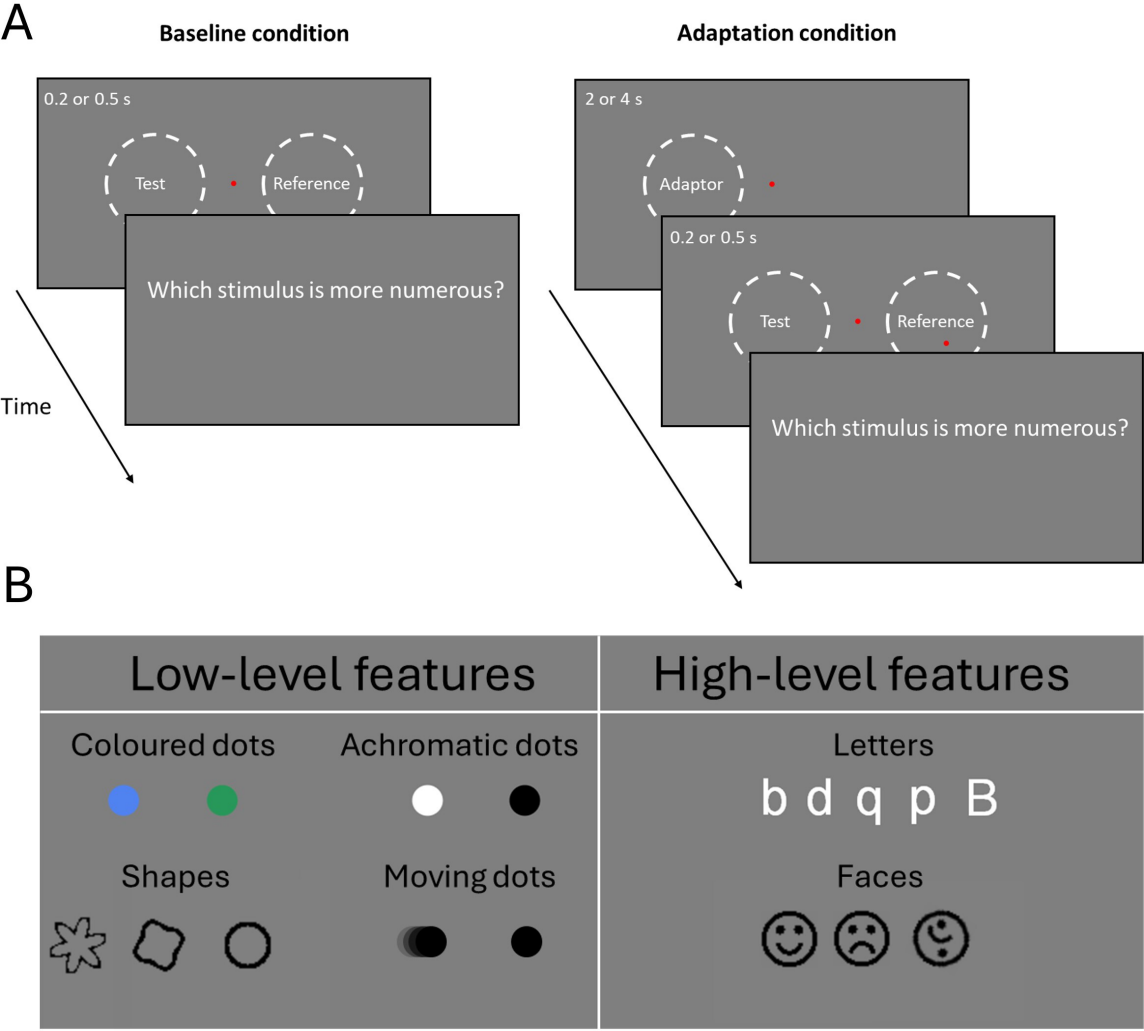
Overview of the experimental design and stimuli. (A) The panels represent the time course of the two conditions, baseline and adaptation. (B) Examples of the stimuli used across the six experiments.

We used a two-alternative forced-choice method (2AFC) to estimate participants’ perceived numerosity, separately in baseline and adaptation conditions (figure 1). Stimuli were arrays of non-overlapping items drawn within a virtual circle (diameter: 8 deg), displayed briefly (200 or 500 ms, depending on the experiment) and simultaneously to the left or to the right of a central fixation point (eccentricity: 10°), on a mid-grey background. One of the two arrays was the reference with numerosity fixed at 24 items, while the other stimulus varied in numerosity for each trial between 10 and 60 or 12 and 48 depending on the experiment. In the adaptation condition, the reference and test stimuli were preceded by an adaptor which was presented for a longer period (2 or 4 s, depending on the experiment) in the same position as the test and with a fixed higher numerosity (48 or 72 items, depending on the experiment). In the experiments with coloured and achromatic dots and letters, the test and reference stimuli (12–48 items) were presented for 200 ms, while the adaptor stimulus, consisting of 48 items, lasted 2 s. In the experiments with moving dots, faces, and shapes, the test and reference stimuli (12–48 items for moving dots and 10−60 items for faces and shapes) were presented for 500 ms and the adaptor, consisting of 72 items, lasted 4 s. In each trial, regardless of the kind of stimuli exploited, the task for the participants was to indicate, via key press, whether the stimulus on the left or right was the more numerous.

Each session comprised trials in which adaptor and test were identical (congruent condition) and trials in which they differed for a given feature (incongruent condition) with the two conditions presented interleaved. The difference in adaptation magnitude measured in the congruent and incongruent conditions defined the amount of selectivity of numerosity adaptation. Below is a detailed description of the stimuli used in each of the six experiments.

Experiment 1. Colour. 20 participants were presented with arrays of coloured dots (diameter: 0.4°). In both the baseline and adaptation conditions, the colour of the test and reference stimuli randomly varied between blue (RGB code: 80 130 240) and green (RGB code: 40 152 90), while the colour of the adaptor was kept fixed (blue).

Experiment 2. Luminance. 20 participants were presented with arrays of black and white dots (diameter: 0.4°). The colour of test and reference randomly varied between black and white, while the colour of the adaptor was fixed to white. In the adaptation condition, in order to avoid visual aftereffects elicited by the prolonged presentation of the white adaptor, a full-screen mask composed of randomly arranged black and white squares (0.2 × 0.2°), was briefly (20 ms) presented soon after the adaptor and before the presentation of the test and reference.

Experiment 3. Shape. 20 participants were presented with arrays of three different shapes. One of the shapes was a circle with a diameter of 0.7° while the other two shapes were obtained by sinusoidally modulating the radius of the circle over 360°: when the modulation was 0.1° in depth and repeated four times, the patches resembled a distorted square while with a 0.3° in depth modulation repeated six times we obtained a stimulus resembling a flower. Importantly, because the positive and negative modulation of the radius were defined by a symmetric sinusoidal profile, these were identical so the perimeter and the area of all the patches were perfectly matched despite their different appearances. In each trial half of the stimuli were white and half black to match the average luminance stimuli with that of the mid-gray background.

Experiment 4. Motion. 20 participants were presented with arrays of moving and static dots (diameter: 0.5°), half white and half black. In the baseline condition, dots in the test and reference stimuli were either static or moving, while the adaptor always consisted of an array of moving dots. All dots moved within an invisible area of 8° diameter at a constant speed of 4° s^−1^. They moved in straight lines and, when colliding with other dots or with the boundaries of the conscription area, they bounced appropriately (obeying the laws of physics).

Experiment 5. Letters. 26 participants were presented with arrays of different letters. For the test and the reference stimuli, we used letters ‘b’, ‘p’, ‘d’, ‘q’ and ‘B’, while the adaptor only included ‘b’s. All letters were matched in size and subtended around 1.2°.

Experiment 6. Faces. 20 participants were presented with arrays of stylized faces (smileys). Test and reference randomly varied between smiling faces, sad faces and scrambled faces, while the adaptor always consisted of an array of smiling faces. Each stimulus subtended 0.8° by 0.8° and, in each display, half of the faces were white and half black to match as average luminance the mid gray background.

In the experiments including shapes, letters and faces, we added a secondary task to ensure participants were capable of discriminating the different stimuli from each other: in one-third of the trials (chosen randomly), after the response to indicate the most numerous stimulus, participants were required to also indicate—via key press—what class of stimuli (in terms of colour, shape and letter) they have been presented with in that trial.

Stimuli were generated and presented with PsychToolbox 3.0.16 routines [41] for Matlab (Ver. R2021b, Mathworks Inc, http://mathworks.com).

#### (ii) Data analysis

Data were analysed separately for each participant and experiment. For each condition, we plotted the percentage of trials in which the test stimulus was perceived more numerous than the reference as a function of tested numerosities and interpolated the data with a best-fitting cumulative Gaussian function. The 50% point of the function defines the point of subjective equality (PSE), the physical numerosity at which the test stimulus was perceptually matched to the reference. We calculated the adaptation effect by subtracting, for each participant, the PSE for the adaptation condition from the PSE measured in baseline (no adaptation) and normalizing this value by the latter.


$$Adaptation\;Effect\left(\%\right)=\frac{PSE\;Adapt\;-PSE\;Base\;}{PSE\;Base}*100.$$

For experiments involving shapes, letters and faces, we also calculated the percentage of correct responses in the secondary task wherein participants indicated the stimulus identity.

Data were analysed with *t*-tests, Repeated-Measures ANOVAs and Bonferroni corrected post-hoc *t*-tests. Bayes factors are Log10 Bayes factors (LogBF10), interpreted as providing substantial (0.5−1), strong (1–2) or decisive (> 2) evidence in favour of the alternative hypothesis. Effect sizes (*η*^2^ or Cohen’s *d*) were also reported. Statistical analyses were performed using JASP (version 0.16.1, The JASP Team 2022, https://jasp-stats.org/).

### (b) Image dissimilarity analysis

To achieve an objective parameter with which to assess the similarity among the stimuli used in the six experiments, we calculated the image dissimilarity between all items exploited as adaptors and tests. To this aim, we used the image dissimilarity toolbox developed by Seibert and Leeds using the Gabor Filterbank method [42]. The distance matrix correlations resulting from the Gabor filter bank model were correlated with the adaptation difference between the congruent condition and the incongruent conditions, across all experiments. The correlation was reported using the Spearman correlation coefficient (rho).

## 3. Results

**Figure 2.**
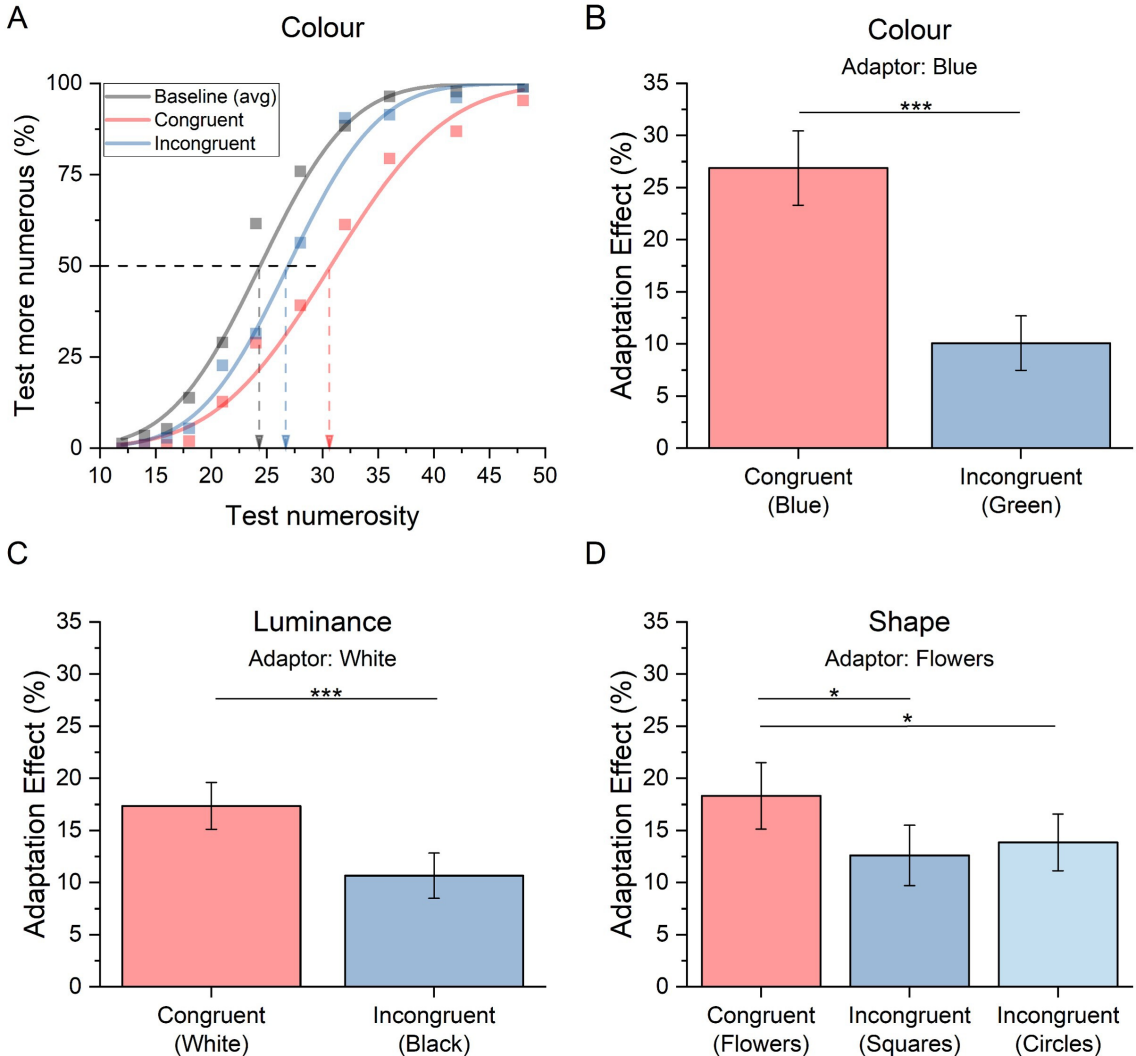
Numerosity adaptation effect measured in the experiments manipulating colour, luminance or shape. (A) Percentage of trials where the test appeared more numerous than the reference as a function of test numerosity. The grey curve shows the aggregate psychometric function measured in baseline while the red and blue curves indicate adaptation for the congruent and incongruent conditions, respectively. The vertical dashed arrows show the estimates of the PSE in each condition. (B–D) The bar graphs show the mean adaptation effect measured in the congruent (red) and the incongruent (blue) conditions when the manipulating items’ colour (B), luminance (C) and shape (D). Error bars are s.e.m.

We first investigated the selectivity for numerosity adaptation due to colour with the aim of replicating the finding by Grasso *et al.* [39]. Participants were adapted to arrays of blue dots and then discriminated which of two simultaneously presented arrays displayed, one on the left and one on the right was more numerous. The condition was congruent when test and reference dots matched the adapter colour (blue) and incongruent when displayed in green. Figure 2A shows the psychometric functions averaged across participants for numerosity discrimination separately for baseline (no adaptation) and adaptation conditions. As the PSE measured in the two baseline conditions, one comprising only blue and the other only green dots, was not significantly different (*t*(19) = −0.49, *p* = 0.63), we pooled these data together to yield a single baseline condition (black curve). Psychometric functions in both adaptation conditions were shifted rightwards relative to baseline, indicating a reduction in perceived numerosity. Importantly, the red curve shows the condition where test and reference matched the adaptor for colour (congruent condition) while the blue curve shows the condition where test and reference had a different colour from the adaptor (incongruent condition). The larger rightward shift of the red psychometric curve indicates a larger adaptation effect when the adapting and adapted stimuli were identical (congruent condition).

Adaptation effects were highly significant in both the congruent and incongruent conditions (both *t*-tests *p* < 0.001). A paired sample *t*‐test confirmed that the percentage of adaptation effect was significantly larger in the congruent compared to the incongruent condition (*t*(19) = 5.61, *p* < 0.001; Cohen’s *d* = 1.3; LogBF10 = 3.1; figure 2B), replicating previous results by Grasso *et al.* [39].

Next, we investigated whether numerosity adaptation selectivity extends to perceptual features other than colour and measured the adaptation magnitude for clouds of achromatic dots. The result (figure 2C) showed a similar trend to that obtained for the manipulation of colour, that is, underestimation in both adaptation conditions and a greater adaptation effect in the congruent condition (adapting and adapted stimuli were all white) compared to the incongruent condition (white adaptor and black test and reference). Adaptation effects in both the congruent and incongruent conditions were highly significant (both *t*-tests *p* < 0.001). A paired sample *t*‐test confirmed that the difference between the adaptation effects was significant (*t*(19) = 3.9, *p* = 0.001; Cohen’s *d* = 0.9; LogBF10 = 1.6) extending the selectivity of numerosity adaptation to luminance polarity congruency.

In experiment 3, we aimed to establish whether the selectivity of numerosity adaptation extended also to another feature typically used to determine object’s identity: its shape. In the congruent condition, the shape of the test and reference stimuli matched that of the adaptor (‘flower shape’), while in the incongruent conditions, they resembled either a square or a circle. Figure 2D shows that, again, the adaptation effect was present across all adaptation conditions and stronger for the congruent compared to the incongruent condition. Adaptation effects in all conditions were significant (all *t*-tests *p* < 0.001). A repeated-measure ANOVA revealed a significant effect of item shape on adaptation (*F*(2,38)=4.3, *p* = 0.02, *η*^2^ = 0.19). Post-hoc *t*-tests revealed that the two incongruent conditions both differed from the congruent condition (squares: *t*(19) =2.6, p_bonf_ = 0.043, Cohen’s *d* = 0.50; circles: *t*(19) =2.5, *p*_bonf_ = 0.047, Cohen’s *d* = 0.50), but not from each other (*t*(19) =−0.04, *p*_bonf_ = 1, Cohen’s *d*= − 0.01).

**Figure 3.**
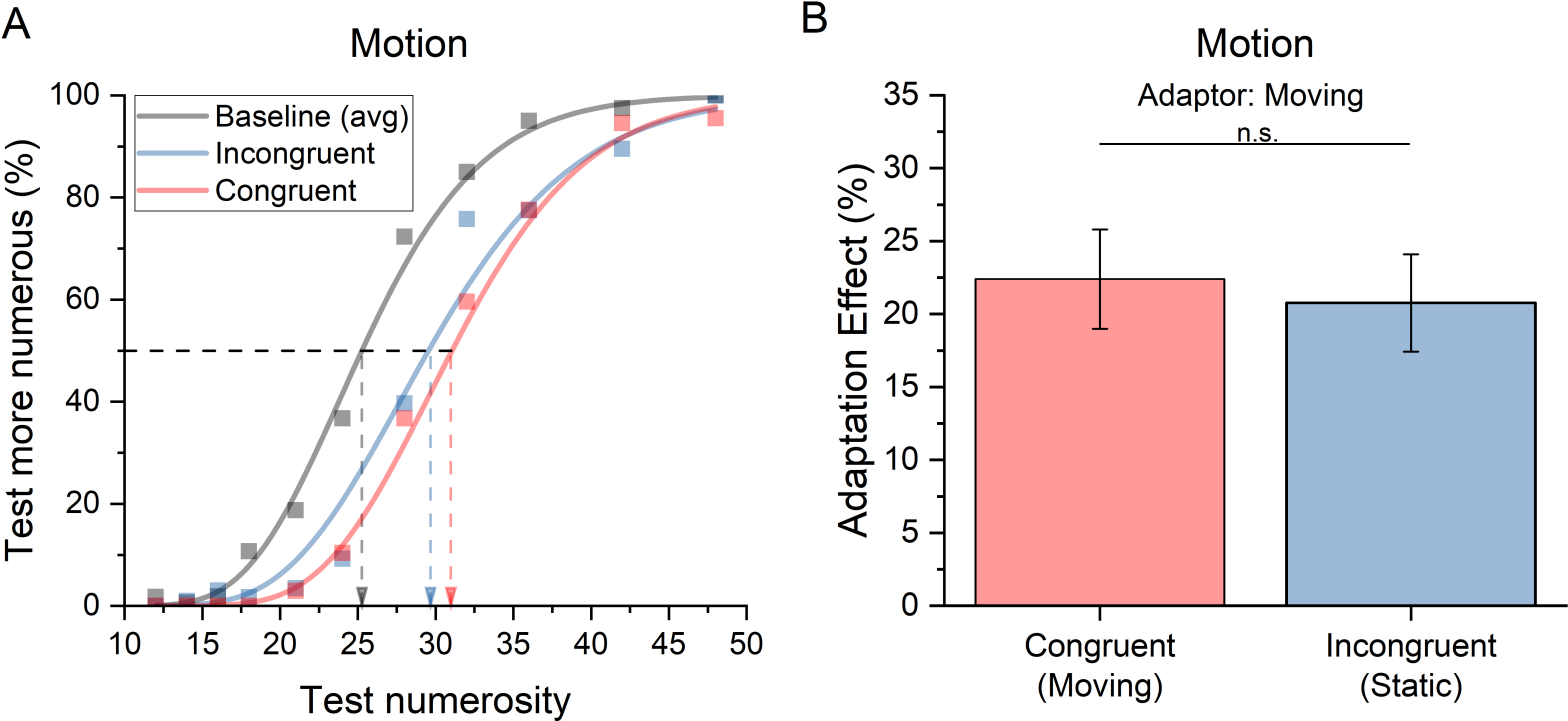
Numerosity adaptation effect measured in the experiment manipulating items’ motion profile. (A) Psychometric functions and (B) bar graph interpretation as in figure 2.

Interestingly, while colour, luminance and shape can define objects’ identity, their change between adapting and adapted stimuli also introduced a ‘novelty effect’ that might be able to disrupt part of the adaptation effect. To assess whether stimuli identity or novelty determined the reduction in adaptation effect in the incongruent condition, we modulated the congruency between adapting and test/reference stimuli in terms of their motion (moving versus still stimuli). If numerosity adaptation selectivity depends on stimulus identity, we expected the same adaptation effect regardless of the motion congruency as it is more common for an object to change position over time than change its colour or shape. Figure 3A shows the psychometric functions, averaged across participants, for conditions with adapting displays of moving dot clouds, and with moving (red) or static (blue) test and reference dots. In the baseline condition (black curve), data for static or moving dots were pooled, since there was no difference in numerosity perception (PSE, *t*(19)=0.7, *p* = 0.5)). The rightward shift of the blue and red psychometric curves indicates a robust numerosity adaptation effect. However, both curves are rather superimposed, suggesting that adaptation magnitude was not affected by motion congruency between test and adaptor. The adaptation effects were present in both conditions (both *p*<  0.001), however in this case the paired sample *t*‐test showed that there was no significant difference between adaptation in the congruent and incongruent conditions (*t*(19) = 0.50, *p* = 0.6; Cohen’s *d* = 0.11; LogBF10 = −0.60), indicating that adapting to moving stimuli distorts the perceived numerosity of both moving and still stimuli to the same extent (figure 3B).

**Figure 4.**
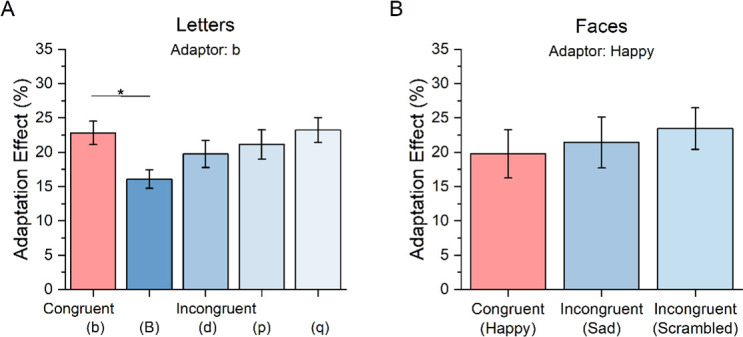
Numerosity adaptation effects measured in the experiment manipulating high-level features. Bar graphs for (A) letter and (B) face stimuli should be interpreted as in figure 2.

Given that all previous experiments suggest that the congruency of stimuli identity between adapting and adapted stimuli might play a key role in defining the magnitude of numerosity adaptation, we designed an additional experiment to test whether the same applied when items’ identification prompts a high-level, linguistic process. By means of simple spatial rotations of the very same shape, we created different letters (i.e. p to b). By using letters, it is possible to create conditions wherein minimal changes in the low-level characteristics of stimuli result in different high-level linguistic meanings. Moreover, as the same letter can be written in different formats, either capital or lower-case, it is possible to achieve bold changes in the shape of the stimuli while maintaining the same high-level linguistic meaning (or identity). We thus defined a congruent condition in which the test and reference were identical to the adaptor (‘b’). Then we designed three incongruent conditions in which the high-level linguistic change was maximized (by the letter change) while keeping minimal the alteration of stimuli low-level features (‘d’, ‘p’, ‘q’). Finally, we devised an additional incongruent condition in which changes in the low-level features were maximized, but the linguistic identity was maintained congruent between test and reference (‘B’) and the adaptor (‘b’). As shown in figure 4A, a strong and robust adaptation effect was observed across all conditions, irrespective of what kind of letter was displayed in lower-case (*p* < 0.001 for all *t*-tests). However, adaptation was remarkably decreased when the test stimulus contained ‘B’s. A repeated-measure ANOVA revealed an effect of the letter on adaptation (*F*(4,25) = 4.9, *p* = 0.001, *η*^2^ = 0.17). Specifically, post hoc t-tests indicated that only when the incongruent condition consisted in a case/shape change (from ‘b’ to ‘B’), the adaptation effect was lower than the congruent condition (*t*(25) =3.7, *p*_bonf_ = 0.004, Cohen’s *d* = 0.75), while in all other conditions, the adaptation magnitude was the same (all *p*-values not significant). This result suggests that adaptation selectivity is less determined by the high-level linguistic meaning (i.e. letter identity) than by the low-level changes of the stimuli features such as the changes in shape induced by changing the letters’ case.

Finally, we used ecological stimuli containing both global and local information in a sixth experiment by adapting and testing participants with stylized representations of faces. In the congruent condition, test and reference were identical to the adaptor (smiling faces), while in the incongruent condition test and reference stimuli consisted either of ‘sad faces’, achieved by rotating upside down the mouth, or ‘scrambled faces’ in which the internal elements (mouth, nose and eyes) were rearranged from their canonical positions. Figure 4C shows there were consistent adaptation aftereffects in all conditions (all *p*< 0.001), but there was no difference across conditions (repeated-measure ANOVA; *F*(2,38)=0.55, *p* = 0.58, *η*^2^ = 0.03). Therefore, when stimuli identity was just defined by changes of local elements while the same global configuration remained the same (faces outline), no categorization of the stimuli takes place to yield selective effects of numerosity adaptation.

**Figure 5.**
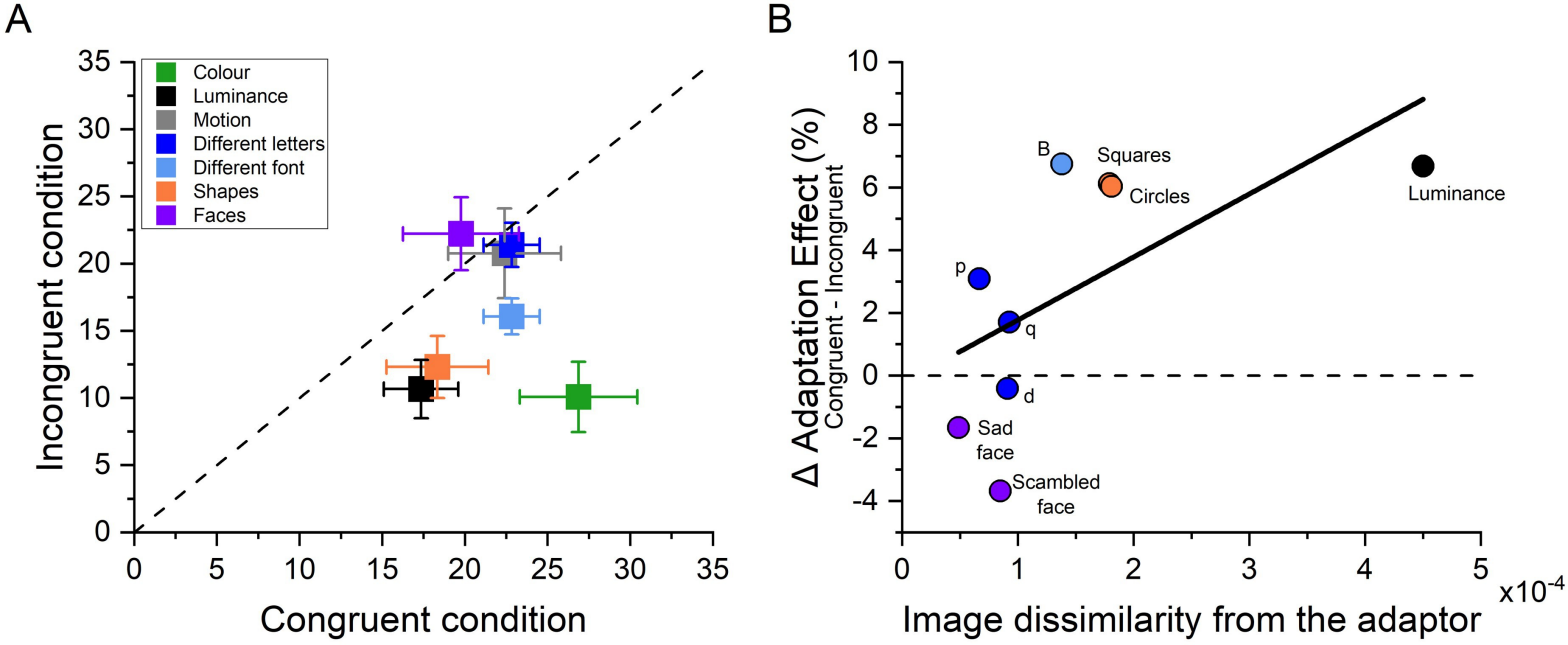
Summary of the adaptation effects measured across experiments. (A) Scatter plot of adaptation effects in the congruent and incongruent conditions, across experiments. Datapoints below the bisector indicate stronger adaptation effect in the congruent compared to the incongruent condition. (B) Correlation between the image dissimilarity and the adaptation effect difference between the congruent and incongruent conditions. The dashed horizontal line indicates the same adaptation effect between the congruent and incongruent conditions.

Figure 5A summarizes the results of all six experiments. Across all experiments we found a significant adaptation effect also for the incongruent conditions, suggesting a consistent part of the numerosity adaptation aftereffects occurs independently of the characteristics of the displayed items. This suggests that stimuli categorization might modulate but not completely account for numerosity adaptation. However, figure 5A also clearly shows that when changes between adaptors and test stimuli regarded low-level features (e.g. colour, luminance, shape or case) a significant reduction of the adaptation effects in the incongruent conditions compared to the congruent condition is observed. Intriguingly, such a selective effect did not hold true in the case of motion as adapting to moving stimuli also robustly distorted numerosity perception of static stimuli. Similarly, the congruency between adaptor and test stimuli did not play a significant role when stimuli were categorized based on high-level features (e.g. faces or letters). To control for item detectability, we added an additional task for the participants in three experiments (shapes, letters and faces) where the stimuli were more complex. In one-third of the trials, participants were required to indicate what kind of item was displayed after having indicated the more numerous stimulus. Accuracy in this secondary task was almost perfect in all experiments, with the percentage of correct response around 90–95% in all conditions.

Even though the observers were capable of identifying the elements included in the test and reference, there is still the possibility that some feature changes were just more salient than others, with the most striking feature changes prompting a more efficient items categorization that, in turn, might have affected numerosity adaptation selectivity. To test this possibility, we calculated the image dissimilarity between adaptor and test stimuli by using a model for the primary visual cortex and correlated the representational dissimilarity matrix resulting from this model with the difference of the adaptation magnitude between the congruent and the incongruent conditions.

Figure 5B shows that as image dissimilarity between the adaptor and test stimuli increased so did the difference between congruent and incongruent adaptation effects (*ρ* = 0.75 *p* = 0.025), pointing at image dissimilarity as a possible key factor in determining the strength of the adaptation effect.

## 4. Discussion

In the current study, we found that numerosity adaptation is a flexible mechanism. Despite the fact that adaptation aftereffects have been observed across a wide variety of different conditions, and regardless of the kind of stimuli used as adaptor or test, our experiments clearly demonstrate that, under some circumstances, numerosity adaptation is significantly larger when the adapting and test stimuli are matched for non-numerical low-level features. For example, numerosity underestimation following adaptation to high numerosity was larger when the test matched the adaptor for colour, luminance or shape compared to when the stimuli differed. Stimulus novelty alone was not sufficient to explain the selectivity of numerosity adaptation, as adaptation to moving stimuli also affected the perceived numerosity of still stimuli, despite the change in the motion profile being very salient. This result seems to suggest that adaptation selectivity might arise from a classification process capable of categorizing the adaptor and test stimuli, with the largest adaptation aftereffects occurring when stimuli shared the same features. Interestingly, this would set the similarity between adaptation and another contextual effect, serial dependency, in which the perception of a stimulus in any given moment is biased to look more similar to that presented a little earlier [43,44]. However, not all the differences between adapting and adapted stimuli were able to attenuate adaptation effects. For example, no reduction in adaptation was observed when moving stimuli were used to adapt the perceived numerosity of still stimuli, despite the striking change in the stimuli’s motion profile. More, in numerosity tasks in which letters were used as stimuli, simple spatial rotations of the same visual pattern yielding different linguistic meanings (different letters) failed to induce adaptation selectivity. On the contrary, keeping the letter the same but changing the format (from lower to uppercase) provided a clear reduction of adaptation aftereffects, indicating that differences on low-level features of the stimuli are capable of overruling congruency at the linguistic level. These results clearly suggest that stimuli identity affects the magnitude of numerosity adaptation just when it is defined by low-level features, not high-level linguistic meaning. Moreover, when stimuli were matched for global information (outlines of stylized faces) but differed for local information (position of the faces components) no adaptation selectivity was found, indicating that the stimuli categorization process bound to numerosity mechanisms might just be able to make a coarse analysis of items’ features. Taken together, our results reveal that the magnitude of adaptation aftereffects is dependent on the low-level image dissimilarity between adaptor and test with such dissimilarity being quantitatively estimated by a model simulating V1 information processing.

It is interesting to note that our results suggest that numerosity perception operates in parallel for different groups of objects and that the perceived numerosity of each group can be independently adapted to some extent. From an ecological perspective, it would be advantageous to form different and selective representations of the number of target objects in a visual scene. Importantly, not all low-level salient changes might be relevant: an object may change its position, but it rarely changes its physical properties such as colour or shape. The adaptation selectivity observed for colour, luminance and shape, but not motion, suggests that numerosity perception is sensitive to salient environmental low-level features that allow us to segregate objects into categories. Numerosity mechanisms might be sensitive to constancies in these features which can be susceptible to selective adaptation. This is in line with previous evidence showing that we can simultaneously keep track of numerosity up to three different subsets of items defined by colour [35].

The present results are particularly important not only because they help clarify how the categorization process in numerosity perception works, but also because they contribute significantly to the ongoing debate over the theoretical interpretation of numerosity adaptation aftereffects. Recently, Yousif *et al.* [36] proposed that numerosity adaptation may not result from changes in the encoding of item quantity in the test (adapted) stimulus—that is, a genuine effect on perceived numerosity. Instead, their ‘old news’ theory suggests that changes in perceived numerosity after adaptation might arise from differences in the saliency of individual elements in the test stimuli, driven by feature changes at the item level [36,37] (but see also [38]). Our data play a crucial role in this dispute. On the one hand, they emphasize the importance of low-level stimulus characteristics in numerosity adaptation; on the other hand, they suggest that stimuli identity defined by low-level features can affect numerosity adaptation magnitude, rather than account for the whole phenomenon. Indeed, in all six conditions examined in this study, we observed a significant amount of numerosity adaptation, even when the adapting and test stimuli were completely different. This implies that a significant portion of the perceptual distortions caused by adaptation cannot be solely attributed to the characteristics of individual items but must involve the numerosity of the item set as a whole. At the same time, since item identity, especially when defined by low-level features, significantly reduced the magnitude of numerosity adaptation in certain conditions, we now have evidence that stimulus categorization, bound to stimulus identity, occurs before adaptation could impact on numerosity perception. Furthermore, in the ‘old news’ hypothesis, the role of novelty in the display (when test stimuli differed from adapters) was somewhat confounded with changes that induced shifts in item identity (see [36,37]). Here, we successfully characterized what are the most important features defining items’ identity in the process of numerosity adaptation.

Further evidence suggesting that numerosity mechanisms operate on segregated and categorized visual items comes from studies showing that various grouping cues can alter numerosity perception. The precision of our numerical estimates increases when multiple items can be segregated into small groups, a phenomenon called ‘groupitizing’ [45–49]. When items are spatially grouped by connectedness (even via illusory connections) or by symmetric cues, perceived numerosity is robustly underestimated with such bias increasing linearly if multiple grouping cues are used at once, suggesting that numerosity mechanisms process both, overall numerosity and number of groups [7,22,50–58]. More, the influence of grouping cues on numerosity perception is much reduced under dual tasks, suggesting that the grouping process requires attention [48,59,60]. Neuroimaging studies further corroborate this, revealing that grouping cues modulate EEG components typically associated with visuo-spatial attention [46]; see reviews by [61,62] and the pattern of activity of areas starting from V3 and beyond [63,64]. By showing that numerosity adaptation selectively impacts different categories of items, the current study suggests that it might occur after grouping or feature bounding, presumably at a relatively late attention-dependent stage. This is in line with previous studies showing that numerosity adaptation operates on perceived rather than physical numerosity when the two differ due to perceptual grouping [22] and that the availability of visuospatial attention during the adaptation period crucially determines the adaptation aftereffects [23,24].

Clearly, grouping cues have to be sufficiently salient to be perceptually noticeable. Differently from what has been found for density adaptation [65,66], in a previous paper Burr and Ross [20] did not find numerosity adaptation selectivity to orientation and size changes between adaptor and test. There are at least two explanations for this observation. First, size and orientation changes may not necessarily correspond to changes in object identity: the same object appears larger when it approaches the observer and can change orientation. An alternative explanation however is that in the experiment by Burr & Ross [20], the changes were not sufficiently salient to be easily discriminated in the peripheral vision. In the current study, we demonstrated that detectability of stimulus changes is indeed a key factor in determining the strength of the adaptation effect, by showing that it can be predicted by image dissimilarity. Future studies should better investigate how size and orientation changes impact the adaptation selectivity.

The reduced discriminability of stimulus changes might in part explain why adaptation selectivity did not occur when adaptor and test differed for high-level features. Adapting to the letter ‘b’ generalized to all the other lower-case letters and adapting to stylized happy faces generalized to both sad and scrambled faces. If we accept to conceive lower-case letters not as letters, but as the same shape differently oriented, then the current findings nicely align with those of Burr & Ross [20] suggesting that adaptation aftereffects are not specific for orientation. However, the dissimilarity between the adaptor and test stimuli in these cases was very low and likely explains the generalization of adaptation. Nevertheless, the fact that adapting to the letter ‘b’ did not generalize to the capital ‘B’ suggests that the low-level shape change prevailed on letter identity in determining the adaptation selectivity or that non-ecological culturally mediated stimuli, such as letters, might not influence numerosity perception. For now, we can conclude that, at least with the stimuli currently used, the low-level feature (shape/font) changes prevailed on the high-level feature changes in driving the adaptation selectivity.

The fact that the selectivity of the adaptation effect can be predicted by image dissimilarity estimated by a simple model of V1 does not necessarily mean that adaptation occurs at this level. Indeed, image dissimilarity calculated with this model also correlated with activity in many areas along the ventral and dorsal stream [42]. Beside the earliest fMRI habituation studies reporting distance-dependent signal release from adaptation in the IPS, later studies using MVPA and pRF found that numerosity adaptation changed the pattern of number-related activity read out from parietal areas [21] as well as the preferred numerosity within several numerosity maps located across the dorsal and ventral pathways [27]. Furthermore, a recent EEG study also revealed that numerosity adaptation selectively attenuates the amplitude of P2p [39], a component closely bounded to the activation of posterior parietal cortex [67–70].

Using an habituation paradigm, one seminal study by Izard *et al.* [3] in human infants found that shape deviants modulated the ERP responses in ventral temporal areas, whereas number deviants modulated the signal in a right parieto-prefrontal network. Importantly however, the authors observed that number of deviants modulated the activity in temporal regions as well. Specifically, they found an antagonistic relationship where number deviants decreased activation in the left anterior temporal regions responsive to object deviants and increased activation in the same region in the right hemisphere, suggesting that object shape (or identity) and numerosity might interact at this level. While mounting evidence shows that the numerical information is also represented along the ventral pathway [71–73] future studies should further investigate where adaptation to numerosity and object identity interact.

It can be argued that the reported effects are similar to contingent aftereffects, reported for several visual features, including perception of density [65,66,74–76]. More specifically, Durgin and collaborators reported a series of evidence that adaptation to dense patterns can be contingently tuned to other low-level features such as orientation and background colour [66,74]. Contingent aftereffects are often thought to arise from features conjunction occurring at early stages, and the fact that, for example, the McCollough effect depends on physical (rather than on perceived) colour is taken in support of this view [74,77]. As a result, it is tempting to assume that also the colour-selective numerosity adaptation may be subserved by similar interactions occurring before stimulus segregation into categories. However, beside the major methodological differences between studies on contingent aftereffects and the present work, it is important to recall that in the paper of Grasso and collaborators [25], the authors showed that the effect was mainly bounded to the perceived (rather than the physical) colour of the set as number adaptation was evident also when the test was perceived having the same colour of the adaptor although being physically different. This is in stark contrast with the contingent aftereffect elicited by coloured background on texture density elements (experiment 3) [74]. In our view, this result speaks about a higher level of interaction between the encoding of number of elements in a set and their colour-dependent identity, a mechanism that might involve the activity of higher-level cortical regions implicated in both numerical coding and categorization of objects through their colour [78].

In conclusion, our findings indicate that numerosity perception can be modulated by contextual effects with previous stimuli capable of distorting numerosity estimates of the following one presented in the same location. Part of the magnitude of these adaptation aftereffects turned out to be selective for salient non-numerical low-level features likely providing cues to define objects’ identity. The results also suggest that numerosity adaptation is likely to occur at relatively high processing levels and that it is influenced by other processes relevant to numerosity perception, including object segmentation and grouping.

## Data Availability

The dataset generated with this study has been deposited on Dryad [79].
